# *N*-Methyl-*N*-phenyl-2-(quinolin-8-yl­oxy)acetamide monohydrate

**DOI:** 10.1107/S1600536811017181

**Published:** 2011-05-14

**Authors:** Li-Hua Zhi, Wei-Na Wu, Shu Li, Yun-Long Li, Xiao-Dong Cai

**Affiliations:** a Department of Physics and Chemistry, Henan Polytechnic University, Jiaozuo 454000, People’s Republic of China

## Abstract

In the title compound, C_18_H_16_N_2_O_2_·H_2_O, the dihedral angle between the quinoline ring system and the benzene ring is 87.19 (8)°. In the crystal, water mol­ecules are linked to acetamide mol­ecules *via* inter­molecular O—H⋯N and O—H⋯O hydrogen bonds.

## Related literature

For the luminescent properties of lanthanide complexes with amide-type ligands, see: Li *et al.* (2003 ▶); Wu *et al.* (2008 ▶). For the synthesis of 2-chloro-*N*-methyl-*N*-phenyl­acetamide, see: Zhi *et al.* (2011 ▶). For the similar structure of *N*-phenyl-2-(quinolin-8-yl­oxy)acetamide hemihydrate, see: Li *et al.* (2005 ▶).
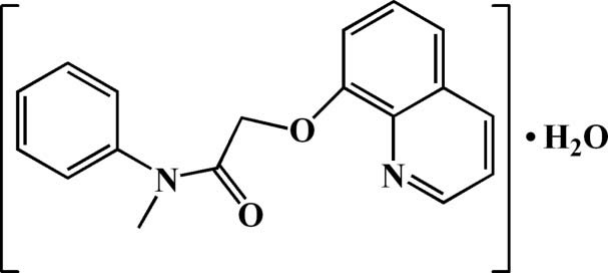


## Experimental

### 

#### Crystal data

C_18_H_16_N_2_O_2_·H_2_O
*M* *_r_* = 310.34
Orthorhombic, 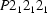

*a* = 6.6028 (8) Å
*b* = 14.9207 (18) Å
*c* = 16.3505 (19) Å
*V* = 1610.8 (3) Å^3^
*Z* = 4
Mo *K*α radiation
μ = 0.09 mm^−1^
*T* = 296 K
0.16 × 0.15 × 0.10 mm

#### Data collection

Bruker APEXII CCD diffractometer
Absorption correction: multi-scan (*SADABS*; Bruker, 2007 ▶) *T*_min_ = 0.986, *T*_max_ = 0.991
10373 measured reflections
3911 independent reflections
3113 reflections with *I* > 2σ(*I*)
*R*_int_ = 0.027

#### Refinement

*R*[*F* ^2^ > 2σ(*F* ^2^)] = 0.039
*wR*(*F* ^2^) = 0.101
*S* = 1.05
3911 reflections
217 parameters
125 restraints
H atoms treated by a mixture of independent and constrained refinement
Δρ_max_ = 0.12 e Å^−3^
Δρ_min_ = −0.14 e Å^−3^

### 

Data collection: *APEX2* (Bruker, 2007 ▶); cell refinement: *SAINT* (Bruker, 2007 ▶); data reduction: *SAINT*; program(s) used to solve structure: *SHELXS97* (Sheldrick, 2008 ▶); program(s) used to refine structure: *SHELXL97* (Sheldrick, 2008 ▶); molecular graphics: *SHELXTL* (Sheldrick, 2008 ▶); software used to prepare material for publication: *SHELXTL*.

## Supplementary Material

Crystal structure: contains datablocks I, global. DOI: 10.1107/S1600536811017181/vm2091sup1.cif

Structure factors: contains datablocks I. DOI: 10.1107/S1600536811017181/vm2091Isup2.hkl

Supplementary material file. DOI: 10.1107/S1600536811017181/vm2091Isup3.cml

Additional supplementary materials:  crystallographic information; 3D view; checkCIF report

## Figures and Tables

**Table 1:** Hydrogen-bond geometry (Å, °)

*D*—H⋯*A*	*D*—H	H⋯*A*	*D*⋯*A*	*D*—H⋯*A*
O1*W*—H1*WA*⋯O2	0.86 (1)	1.97 (1)	2.8249 (19)	178 (3)
O1*W*—H1*WB*⋯N1	0.86 (1)	1.97 (1)	2.831 (2)	176 (3)

**Fig. 1.**
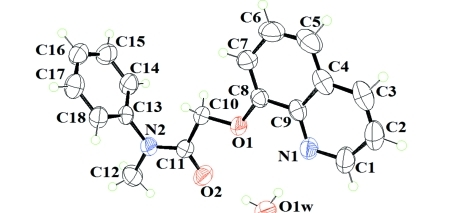
The molecular structure of the title compound with displacement ellipsoids shown at the 50% probability level.

## supplementary crystallographic information

### Comment

The amide type open-chain ligands have attracted much attention mainly because their excellent coordination ability and high selectivity to metal ions. Lanthanide complexes usually exhibit fascinating properties that may have potential applications in biology, medicine, and material science (Li *et al.*, 2003). The luminescent properties of lanthanide complexes with amide type ligands have been investigated in our previous work (Wu *et al.*, 2008). As part of our ongoing studies of the amide type ligands, the title compound was synthesized and characterized by X-ray diffraction.

In the title compound, all bond lengths are comparable with those observed in a similar compound (Li *et al.*, 2005). The dihedral angle between the quinoline ring (N1/C1–C9, r.m.s. deviation 0.0038 Å) and the benzene ring(C13–C18, r.m.s. deviation 0.0049 Å) is 87.19 (8)°. In the crystal structure, solvent water molecules form intermolecular O—H···N and O—H···O hydrogen bonds with acetamide molecules to stabilize the packing (Table 1).

### Experimental

8-Hydroxyquinoline (1.5 g, 10.3 mmol) and anhydrous potassium carbonate (1.6 g, 11.6 mmol)were added to DMF (15 mL), then 2-chloro-*N*-methyl-*N*-phenylacetamide (1.83 g, 10.0 mmol, Zhi *et al.*, 2011) and a small quantity of KI were added. The reaction mixture was stirred for 5 h at 100–110 °C. After cooling down, 150 mL water was added and stirred for 2 h. The precipitate was collected by filtration and washed with water. Recrystallization from EtOH/H_2_O (1:1) gave colorless blocks.

### Refinement

C-bound H atoms were placed in calculated positions (C—H = 0.93 and 0.97 Å) and were included in the refinement in the riding model approximation, with *U*_iso_(H) = 1.2Ueq(C). The water H atoms were located from difference Fourier map calculation and then refined unsing *DFIX* and DANG instruction, with O—H = 0.85Å and *U*_iso_(H) = 1.5Ueq(O). DELU, SIMU and ISOR restraints have been applied on the *U_ij_*-values of the C atoms.

### Crystal data

**Table:**

C_18_H_16_N_2_O_2_·H_2_O	*F*(000) = 656
*M**_r_* = 310.34	*D*_x_ = 1.280 Mg m^−^^3^
Orthorhombic, *P*2_1_2_1_2_1_	Mo *K*α radiation, λ = 0.71073 Å
Hall symbol: P 2ac 2ab	θ = 1.9–28.2°
*a* = 6.6028 (8) Å	µ = 0.09 mm^−^^1^
*b* = 14.9207 (18) Å	*T* = 296 K
*c* = 16.3505 (19) Å	Block, colorless
*V* = 1610.8 (3) Å^3^	0.16 × 0.15 × 0.10 mm
*Z* = 4	

### Data collection

**Table:**

Bruker SMART CCD diffractometer	3911 independent reflections
Radiation source: fine-focus sealed tube	3113 reflections with *I* > 2σ(*I*)
graphite	*R*_int_ = 0.027
φ and ω scans	θ_max_ = 28.2°, θ_min_ = 1.9°
Absorption correction: multi-scan (*SADABS*; Bruker, 2007)	*h* = −8→8
*T*_min_ = 0.986, *T*_max_ = 0.991	*k* = −19→18
10373 measured reflections	*l* = −21→18

### Refinement

**Table:**

Refinement on *F*^2^	Secondary atom site location: difference Fourier map
Least-squares matrix: full	Hydrogen site location: inferred from neighbouring sites
*R*[*F*^2^ > 2σ(*F*^2^)] = 0.039	H atoms treated by a mixture of independent and constrained refinement
*wR*(*F*^2^) = 0.101	*w* = 1/[σ^2^(*F*_o_^2^) + (0.0498*P*)^2^ + 0.0847*P*] where *P* = (*F*_o_^2^ + 2*F*_c_^2^)/3
*S* = 1.05	(Δ/σ)_max_ = 0.005
3911 reflections	Δρ_max_ = 0.12 e Å^−^^3^
217 parameters	Δρ_min_ = −0.14 e Å^−^^3^
125 restraints	Extinction correction: *SHELXL97* (Sheldrick, 2008), Fc^*^=kFc[1+0.001xFc^2^λ^3^/sin(2θ)]^-1/4^
Primary atom site location: structure-invariant direct methods	Extinction coefficient: 0.0132 (19)

### Special details

**Table:**

Geometry. All e.s.d.'s (except the e.s.d. in the dihedral angle between two l.s. planes) are estimated using the full covariance matrix. The cell e.s.d.'s are taken into account individually in the estimation of e.s.d.'s in distances, angles and torsion angles; correlations between e.s.d.'s in cell parameters are only used when they are defined by crystal symmetry. An approximate (isotropic) treatment of cell e.s.d.'s is used for estimating e.s.d.'s involving l.s. planes.
Refinement. Refinement of *F*^2^ against ALL reflections. The weighted *R*-factor *wR* and goodness of fit *S* are based on *F*^2^, conventional *R*-factors *R* are based on *F*, with *F* set to zero for negative *F*^2^. The threshold expression of *F*^2^ > σ(*F*^2^) is used only for calculating *R*-factors(gt) *etc*. and is not relevant to the choice of reflections for refinement. *R*-factors based on *F*^2^ are statistically about twice as large as those based on *F*, and *R*- factors based on ALL data will be even larger.

### Fractional atomic coordinates and isotropic or equivalent isotropic displacement parameters (Å^2^)

**Table:**

	*x*	*y*	*z*	*U*_iso_*/*U*_eq_	
O1	0.80212 (17)	0.10711 (7)	0.15970 (6)	0.0452 (3)	
O2	0.8822 (2)	0.06977 (8)	0.31349 (8)	0.0583 (4)	
C8	0.7564 (3)	0.12177 (10)	0.07926 (9)	0.0420 (4)	
N2	0.6671 (2)	0.17225 (9)	0.36347 (8)	0.0471 (3)	
N1	1.0695 (2)	0.04605 (10)	0.05053 (9)	0.0510 (4)	
C10	0.6699 (3)	0.14635 (11)	0.21760 (9)	0.0440 (4)	
H10A	0.5345	0.1221	0.2113	0.053*	
H10B	0.6637	0.2107	0.2095	0.053*	
C11	0.7500 (2)	0.12557 (10)	0.30174 (10)	0.0419 (3)	
C9	0.9020 (3)	0.08886 (10)	0.02220 (10)	0.0437 (4)	
C13	0.5129 (2)	0.23910 (10)	0.35189 (9)	0.0415 (4)	
C7	0.5874 (3)	0.16515 (12)	0.05232 (12)	0.0552 (5)	
H7	0.4921	0.1860	0.0896	0.066*	
C18	0.5634 (3)	0.32878 (11)	0.35671 (12)	0.0525 (4)	
H18	0.6969	0.3456	0.3665	0.063*	
C4	0.8680 (3)	0.10316 (12)	−0.06202 (11)	0.0574 (5)	
C14	0.3166 (3)	0.21536 (12)	0.33744 (12)	0.0560 (5)	
H14	0.2810	0.1551	0.3349	0.067*	
C17	0.4168 (3)	0.39294 (12)	0.34698 (13)	0.0596 (5)	
H17	0.4515	0.4532	0.3504	0.072*	
C2	1.1823 (4)	0.02794 (15)	−0.08759 (13)	0.0711 (5)	
H2	1.2811	0.0065	−0.1230	0.085*	
C3	1.0166 (4)	0.07026 (13)	−0.11641 (12)	0.0676 (5)	
H3	1.0001	0.0778	−0.1725	0.081*	
C12	0.7333 (4)	0.15493 (14)	0.44699 (11)	0.0660 (5)	
H12A	0.8400	0.1112	0.4465	0.099*	
H12B	0.6214	0.1326	0.4785	0.099*	
H12C	0.7821	0.2095	0.4710	0.099*	
C1	1.2014 (3)	0.01726 (14)	−0.00346 (12)	0.0651 (5)	
H1	1.3157	−0.0124	0.0159	0.078*	
C15	0.1708 (3)	0.28045 (14)	0.32658 (14)	0.0636 (5)	
H15	0.0379	0.2638	0.3153	0.076*	
C16	0.2201 (3)	0.36932 (13)	0.33224 (11)	0.0572 (5)	
H16	0.1210	0.4131	0.3261	0.069*	
C6	0.5576 (4)	0.17827 (13)	−0.03189 (14)	0.0698 (6)	
H6	0.4425	0.2084	−0.0497	0.084*	
C5	0.6923 (4)	0.14804 (14)	−0.08755 (13)	0.0716 (6)	
H5	0.6686	0.1570	−0.1430	0.086*	
O1W	1.2156 (2)	0.02723 (13)	0.21238 (9)	0.0780 (5)	
H1WA	1.116 (3)	0.039 (2)	0.2438 (12)	0.117*	
H1WB	1.175 (4)	0.035 (2)	0.1629 (7)	0.117*	

### Atomic displacement parameters (Å^2^)

**Table:**

	*U*^11^	*U*^22^	*U*^33^	*U*^12^	*U*^13^	*U*^23^
O1	0.0470 (6)	0.0525 (6)	0.0361 (6)	0.0104 (5)	0.0031 (5)	−0.0040 (5)
O2	0.0599 (8)	0.0629 (7)	0.0521 (7)	0.0214 (7)	0.0033 (6)	0.0043 (6)
C8	0.0488 (9)	0.0365 (7)	0.0408 (8)	−0.0012 (7)	−0.0025 (7)	−0.0026 (6)
N2	0.0526 (8)	0.0498 (7)	0.0388 (7)	0.0069 (7)	−0.0004 (6)	−0.0044 (6)
N1	0.0518 (8)	0.0548 (8)	0.0464 (8)	0.0031 (7)	0.0078 (7)	−0.0076 (7)
C10	0.0421 (8)	0.0464 (8)	0.0434 (9)	0.0066 (7)	0.0034 (7)	−0.0065 (6)
C11	0.0407 (8)	0.0403 (7)	0.0447 (8)	−0.0014 (7)	0.0044 (7)	0.0006 (6)
C9	0.0550 (10)	0.0375 (8)	0.0387 (8)	−0.0057 (7)	0.0012 (7)	−0.0039 (6)
C13	0.0455 (9)	0.0432 (8)	0.0359 (8)	0.0022 (7)	0.0068 (7)	−0.0048 (6)
C7	0.0585 (11)	0.0503 (9)	0.0567 (11)	0.0080 (9)	−0.0105 (9)	−0.0038 (8)
C18	0.0482 (9)	0.0485 (9)	0.0609 (11)	−0.0033 (9)	0.0073 (8)	−0.0083 (8)
C4	0.0805 (12)	0.0517 (9)	0.0399 (9)	−0.0131 (9)	0.0012 (8)	−0.0027 (7)
C14	0.0520 (10)	0.0456 (9)	0.0702 (12)	−0.0054 (8)	0.0062 (9)	−0.0068 (8)
C17	0.0673 (12)	0.0426 (9)	0.0688 (12)	−0.0005 (9)	0.0122 (10)	−0.0021 (8)
C2	0.0772 (12)	0.0777 (11)	0.0583 (10)	−0.0052 (11)	0.0226 (10)	−0.0194 (9)
C3	0.0946 (13)	0.0671 (10)	0.0410 (9)	−0.0168 (10)	0.0102 (9)	−0.0082 (8)
C12	0.0814 (14)	0.0756 (12)	0.0409 (9)	0.0124 (11)	−0.0048 (10)	−0.0004 (8)
C1	0.0650 (11)	0.0710 (10)	0.0594 (10)	0.0011 (10)	0.0170 (9)	−0.0157 (8)
C15	0.0460 (10)	0.0669 (12)	0.0779 (13)	0.0010 (9)	0.0040 (10)	−0.0074 (10)
C16	0.0607 (12)	0.0571 (10)	0.0538 (10)	0.0140 (9)	0.0081 (9)	0.0007 (8)
C6	0.0790 (14)	0.0625 (11)	0.0680 (13)	0.0115 (11)	−0.0239 (11)	0.0068 (10)
C5	0.1004 (17)	0.0673 (12)	0.0472 (11)	−0.0026 (12)	−0.0181 (12)	0.0063 (9)
O1W	0.0531 (8)	0.1216 (12)	0.0594 (8)	0.0265 (9)	−0.0078 (7)	−0.0196 (9)

### Geometric parameters (Å, °)

**Table:**

O1—C8	1.3670 (19)	C4—C3	1.412 (3)
O1—C10	1.4148 (19)	C14—C15	1.379 (3)
O2—C11	1.2217 (19)	C14—H14	0.9300
C8—C7	1.363 (2)	C17—C16	1.367 (3)
C8—C9	1.427 (2)	C17—H17	0.9300
N2—C11	1.343 (2)	C2—C3	1.348 (3)
N2—C13	1.438 (2)	C2—C1	1.391 (3)
N2—C12	1.457 (2)	C2—H2	0.9300
N1—C1	1.312 (2)	C3—H3	0.9300
N1—C9	1.358 (2)	C12—H12A	0.9600
C10—C11	1.506 (2)	C12—H12B	0.9600
C10—H10A	0.9700	C12—H12C	0.9600
C10—H10B	0.9700	C1—H1	0.9300
C9—C4	1.412 (2)	C15—C16	1.368 (3)
C13—C14	1.364 (3)	C15—H15	0.9300
C13—C18	1.381 (2)	C16—H16	0.9300
C7—C6	1.405 (3)	C6—C5	1.350 (3)
C7—H7	0.9300	C6—H6	0.9300
C18—C17	1.371 (3)	C5—H5	0.9300
C18—H18	0.9300	O1W—H1WA	0.855 (10)
C4—C5	1.403 (3)	O1W—H1WB	0.860 (10)
			
C8—O1—C10	116.19 (12)	C13—C14—H14	119.9
C7—C8—O1	124.58 (15)	C15—C14—H14	119.9
C7—C8—C9	120.25 (15)	C16—C17—C18	120.76 (17)
O1—C8—C9	115.17 (14)	C16—C17—H17	119.6
C11—N2—C13	123.34 (13)	C18—C17—H17	119.6
C11—N2—C12	119.34 (15)	C3—C2—C1	118.3 (2)
C13—N2—C12	117.32 (14)	C3—C2—H2	120.9
C1—N1—C9	117.70 (16)	C1—C2—H2	120.9
O1—C10—C11	108.01 (13)	C2—C3—C4	120.4 (2)
O1—C10—H10A	110.1	C2—C3—H3	119.8
C11—C10—H10A	110.1	C4—C3—H3	119.8
O1—C10—H10B	110.1	N2—C12—H12A	109.5
C11—C10—H10B	110.1	N2—C12—H12B	109.5
H10A—C10—H10B	108.4	H12A—C12—H12B	109.5
O2—C11—N2	121.78 (15)	N2—C12—H12C	109.5
O2—C11—C10	122.34 (14)	H12A—C12—H12C	109.5
N2—C11—C10	115.88 (14)	H12B—C12—H12C	109.5
N1—C9—C4	122.23 (16)	N1—C1—C2	124.6 (2)
N1—C9—C8	119.17 (14)	N1—C1—H1	117.7
C4—C9—C8	118.59 (17)	C2—C1—H1	117.7
C14—C13—C18	119.42 (16)	C16—C15—C14	120.51 (19)
C14—C13—N2	121.02 (15)	C16—C15—H15	119.7
C18—C13—N2	119.55 (15)	C14—C15—H15	119.7
C8—C7—C6	119.84 (19)	C17—C16—C15	119.19 (19)
C8—C7—H7	120.1	C17—C16—H16	120.4
C6—C7—H7	120.1	C15—C16—H16	120.4
C17—C18—C13	119.93 (17)	C5—C6—C7	121.5 (2)
C17—C18—H18	120.0	C5—C6—H6	119.3
C13—C18—H18	120.0	C7—C6—H6	119.3
C5—C4—C9	119.61 (18)	C6—C5—C4	120.24 (19)
C5—C4—C3	123.58 (19)	C6—C5—H5	119.9
C9—C4—C3	116.8 (2)	C4—C5—H5	119.9
C13—C14—C15	120.17 (17)	H1WA—O1W—H1WB	107.3 (16)
			
C10—O1—C8—C7	−5.0 (2)	C14—C13—C18—C17	0.0 (3)
C10—O1—C8—C9	174.32 (13)	N2—C13—C18—C17	−179.02 (16)
C8—O1—C10—C11	−177.88 (13)	N1—C9—C4—C5	−179.83 (17)
C13—N2—C11—O2	179.28 (15)	C8—C9—C4—C5	−0.7 (2)
C12—N2—C11—O2	−1.2 (2)	N1—C9—C4—C3	0.3 (2)
C13—N2—C11—C10	−0.7 (2)	C8—C9—C4—C3	179.43 (15)
C12—N2—C11—C10	178.81 (16)	C18—C13—C14—C15	0.9 (3)
O1—C10—C11—O2	−12.8 (2)	N2—C13—C14—C15	179.93 (17)
O1—C10—C11—N2	167.15 (14)	C13—C18—C17—C16	−0.2 (3)
C1—N1—C9—C4	−0.3 (2)	C1—C2—C3—C4	0.4 (3)
C1—N1—C9—C8	−179.41 (16)	C5—C4—C3—C2	179.79 (19)
C7—C8—C9—N1	179.84 (15)	C9—C4—C3—C2	−0.4 (3)
O1—C8—C9—N1	0.5 (2)	C9—N1—C1—C2	0.4 (3)
C7—C8—C9—C4	0.7 (2)	C3—C2—C1—N1	−0.4 (3)
O1—C8—C9—C4	−178.61 (14)	C13—C14—C15—C16	−1.7 (3)
C11—N2—C13—C14	77.0 (2)	C18—C17—C16—C15	−0.5 (3)
C12—N2—C13—C14	−102.5 (2)	C14—C15—C16—C17	1.5 (3)
C11—N2—C13—C18	−103.99 (19)	C8—C7—C6—C5	0.6 (3)
C12—N2—C13—C18	76.5 (2)	C7—C6—C5—C4	−0.7 (3)
O1—C8—C7—C6	178.61 (17)	C9—C4—C5—C6	0.7 (3)
C9—C8—C7—C6	−0.7 (3)	C3—C4—C5—C6	−179.47 (19)

### Hydrogen-bond geometry (Å, °)

**Table:**

*D*—H···*A*	*D*—H	H···*A*	*D*···*A*	*D*—H···*A*
O1W—H1WA···O2	0.86 (1)	1.97 (1)	2.8249 (19)	178 (3)
O1W—H1WB···N1	0.86 (1)	1.97 (1)	2.831 (2)	176 (3)
